# Engineered E. coli Nissle 1917 for the reduction of vancomycin‐resistant *Enterococcus* in the intestinal tract

**DOI:** 10.1002/btm2.10107

**Published:** 2018-09-08

**Authors:** Kathryn G. Geldart, Sushma Kommineni, Madeline Forbes, Michael Hayward, Gary M. Dunny, Nita H. Salzman, Yiannis N. Kaznessis

**Affiliations:** ^1^ General Probiotics Inc. St. Paul MN 55114; ^2^ Department of Microbiology and Immunology University of Minnesota Minneapolis MN 55455; ^3^ Merck Cambridge MA 02141; ^4^ Takeda Pharmaceuticals Minneapolis MN 55455; ^5^ Department of Pediatrics Medical College of Wisconsin Milwaukee WI 53226; ^6^ Department of Microbiology and Immunology Medical College of Wisconsin Milwaukee WI 53226

**Keywords:** antimicrobial peptides, antimicrobials, bacteriocins, E. coli Nissle 1917, microbiome engineering, synthetic biology, vancomycin‐resistant enterococcus

## Abstract

Vancomycin‐resistant *Enterococcus* (VRE) poses a serious threat in hospitals where they densely colonize the intestinal tracts of patients. In vulnerable hosts, these pathogens may translocate to the bloodstream and become lethal. The ability to selectively reduce VRE in the intestinal tracts of patients could potentially prevent many of these translocation events and reduce the spread of the pathogen. Herein, we have engineered Escherichia. coli Nissle 1917 to produce and secrete three antimicrobial peptides, Enterocin A, Enterocin B, and Hiracin JM79, to specifically target and kill *Enterococcus.* These peptides exhibited potent activity against both *Enterococcus faecium* and *Enterococcus faecalis*, the two most prominent species responsible for VRE infections. We first discuss the optimization of the system used to express and secrete the peptides. We then show that by simultaneously expressing these peptides, both *E. faecium* and *E. faecalis* were drastically inhibited. We then demonstrate a suppression of the development of resistance when supernatant from the E. coli producer strains was used to treat *E. faecium.* Finally, we tested the efficacy of the probiotic in a VRE colonization model in mice. These studies showed that administration of the engineered probiotic significantly reduced the levels of both *E. faecium* and *E. faecalis* in the feces of male Balb/cJ mice.

## 1 INTRODUCTION

Vancomycin‐resistant enterococci (VRE) are currently the fourth most common cause of death by antibiotic resistant infection in the United States.^1^ Over 20 000 VRE infections occur each year in the United States alone and over 1 in 20 patients infected with VRE die as a direct result of the infection.^1^ These pathogens are considered a “serious threat” by the Centers for Disease Control and Prevention (CDC) because of their frequent high‐level resistance to a wide array of antibiotics, including vancomycin, an antibiotic previously used as a last resort for enterococci infections.^1^

Enterococci have become ubiquitous infectious agents in nosocomial environments, where they act as opportunistic pathogens, due to their resistance to commonly used antibiotics.^2^ The common occurrence of VRE in the native microbiota of patients plays a significant role in the spread of the pathogen.^3^ It is estimated that approximately 10% of intensive care units patients are colonized with VRE upon admission and that another 10% will become colonized during their stay.^4^

Low‐level VRE colonization of the intestines is generally benign but becomes problematic when patients are treated with antibiotics.^5^ Upon treatment, a patient's native gut microbiome is drastically altered and becomes an easily colonizable niche for the resistant enterococci.^6^ In some patients, particularly those with compromised immune systems, the colonized VRE then translocate to other parts of the body, causing potentially lethal infections.^2^ Frequently, even densely colonized patients exhibit no symptoms and act as long‐lasting reservoirs of the pathogen, making it difficult to eradicate the bacteria from the hospital environment.^7^, ^8^ The ability to selectively eliminate VRE from the gastrointestinal (GI) tracts of densely colonized patients, or patients at risk of colonization, could help prevent both lethal translocation events and reduce hospital contamination.^3^, ^6^

Antimicrobial peptides (AMPs) are small peptides, typically less than 100 amino acids naturally produced by many organisms as a first line of defense against invading pathogens.^9^ Over 70 AMPs have been tested and reported to have activity against enterococci.^10^ Bacteriocins are a broad class of AMPs produced by bacterial species and often target other species phylogenetically similar to the producer strain.^11^ A major benefit of the bacteriocins as an antimicrobial agent is their specificity compared to many traditional antibiotics.^12^, ^13^ Because of this specificity, they may offer a means of reducing unwanted pathogenic species, like VRE, from the gut microbiota while causing minimal changes to other potentially protective microbes.^12^

However, a major challenge in using AMPs for internal infections is that they are often readily degraded in the body and thus cannot reach the site of infection in sufficient quantities when orally administered.^14^, ^15^ To overcome this delivery challenge, we are engineering probiotic bacteria that can survive passage through and temporarily reside in the GI tract. Once at the site of infection, they will produce and secrete the AMPs to eliminate the pathogen of interest. After the threat of infection has passed, probiotic administration will be ceased, and depending on the delivery organism selected, the probiotics will be naturally shed from the patient.^16^, ^17^, ^18^

For this application, we have selected *Escherichia coli* Nissle 1917 (EcN) as our delivery organism. EcN is a well‐established probiotic strain that has been used in humans for over a century.^16^, ^19^ EcN has been demonstrated to have numerous health benefits including anti‐inflammatory effects, induction of gut immune defenses, and treatment of both diarrhea and constipation.^19^, ^20^ Importantly, EcN has also been found to strengthen the integrity of the intestinal wall thereby preventing pathogen translocation.^21^

To engineer EcN to deliver AMPs, one must identify both the appropriate peptides and a viable secretion system for those peptides. Recently, an elegant study was published by Hwang et al. in which EcN was engineered to produce the bacteriocin pyocin S5 and release the protein via cell lysis in response to a quorum sensing signal produced by the pathogen *Pseudomonas aeruginosa* .^22^ This mode of release via cell lysis is common for the production of large bacteriocins produced by and targeting Gram‐negative species (ex. colicins and pyocins) but is rarely described for the smaller bacteriocins targeting Gram‐positive species.^11^, ^13^

Several others have described heterologous production and secretion of bacteriocins from Gram‐negative producers using dedicated secretion systems isolated from the original bacteriocin gene clusters.^23^, ^24^, ^25^ Frequently, bacteriocin genetic clusters contain their own dedicated secretion system and heterologous production can be achieved by direct transfer of the cluster into a desired producer strain.^26^, ^27^ However, as mentioned above, most bacteriocins targeting *Enterococcus*, a Gram‐positive genus, will be of Gram‐positive origin. Therefore, due to the vastly different membrane structure of Gram‐positive and Gram‐negative species, it is unlikely that the secretion systems will be functional in the Gram‐negative EcN.^28^ One must then identify a secretion system that is both compatible with EcN and can secrete a variety of Gram‐positive bacteriocins.

We previously developed a peptide expression vector for EcN that employs the Microcin V secretion system to secrete a variety of bacteriocins of Gram‐positive and Gram‐negative origin.^29^ We showed that this modular peptide secretion system (pMPES) could secrete inhibitory concentrations of four bacteriocins with activity against VRE. However, it was expected that significant improvements were needed to make this a viable peptide delivery system for animal studies.

Herein, we describe improvements to the original modular secretion system and we examine EcN as a viable delivery vehicle for three anti‐enteroccocal peptides; Enterocin A, Enterocin B, and Hiracin JM79. We begin by describing optimization of the peptide production system used for EcN. We then test the *in vitro* activity of these systems and demonstrate that this activity is indeed due to the secretion of the peptides. Finally, we test our engineered probiotic in a murine colonization model to evaluate its ability to reduce VRE in the GI tract.

## 2 RESULTS AND DISCUSSION

### 2.1 Plasmid design and construction

Previously we reported on a modular peptide expression system (pMPES) that used the Microcin V secretion system to express and secrete several AMPs targeting intestinal pathogens such as *E. coli*, *Salmonella*, and VRE. The original pMPES contained the entire 9.1 kb fragment originally isolated by Gilson et al. that encompassed the entire Microcin V production cluster.^30^ This original Microcin V production cluster contains four genes required for Microcin V synthesis; *cvaC*, *cvi*, *cvaA*, and *cvaB.* 30 *CvaC* encodes for the Microcin V peptide, *cvi* encodes for the Microcin V immunity gene, and *cvaA* and *cvaB* encode for the secretion machinery.^30^ The specific functions of these secretion genes and their proposed orientations in the membrane have been described by Hwang et al.^31^

**Figure 1:**
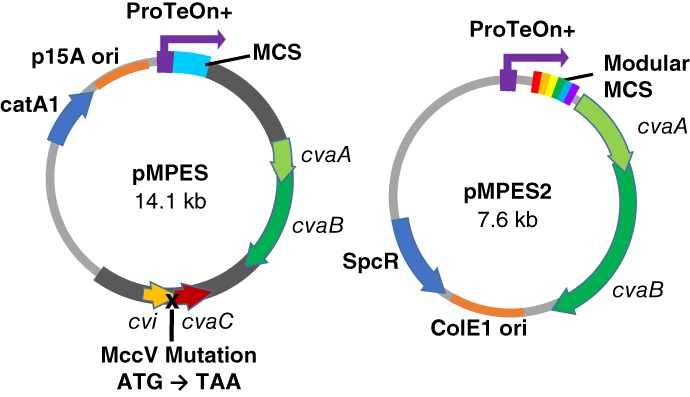
Comparison of the pMPES and pMPES2 vector components. ProTeOn+: Synthetic DNA promoter;cvaA/cvaB: Microcin V section machinery; cvaC: Microcin V peptide (native); cvi: Microcin V immunity protein; MCS: Multiple cloning site, SpcR: Spectinomycin resistance gene; catA1: Chloramphenicol resistance gene. Dark gray region on pMPES indicates uncharacterized or unnecessary genetic information that was removed in the creation of pMPES2

To create pMPES2, we first sought to reduce unnecessary genetic components to create a more well‐defined, less cumbersome vector. The two genes, *cvaA* and *cvaB*, are the only reported plasmid‐derived components required for Microcin V secretion.^30^, ^32^ We thus anticipated that by isolating these genes and their promoter regions, we could attain peptide secretion. A 3.6 kb fragment containing only the *cvaA* and *cvaB* genes along with ~150 basepairs up and downstream of the genes was isolated from the native Microcin V production cluster. The 3.6 kb fragment was then inserted into a backbone containing the ColEI origin of replication instead of the original p15A origin of replication. We note that the p15A origin of replication typically results in a lower copy number.^33^ Figure 1 shows the genetic maps of pMPES and pMPES2.

The second primary modification incorporated in pMPES2 was the addition of an optimized, modular molecular cloning site. We aimed to make single and multiple peptide insertion rapid and standardized. This minimizes the time required for design and it makes comparisons between variables more controllable.

In our modular system, we added a standard optimized, ribosomal binding site (RBS) to the 5′ end of each peptide gene. On the 3′ end, we added a standard primer binding site (PBS). We then designed a set of primers containing overlap regions that could be used to amplify any peptide flanked by the standard RBS and PBS. The overlaps incorporated by the primers enable individual or multiple peptides (up to five) to be simultaneously assembled into pMPES2 using Gibson assembly. This method is extremely beneficial when different combinations and orders of peptides are to be tested. Figure S1 (Supporting Information) depicts this modular assembly method.

The standard RBS used here was specifically optimized for this application using the RBS Calculator.^34^, ^35^ It was previously found that while the original pMPES was functional, its activity could be drastically improved by minor changes in the RBS.^29^ It has been shown that the translation efficiency is significantly impacted by the genetic sequence immediately up and downstream of the RBS.^36^, ^37^ For the upstream sequence, we used the sequence produced by the the Salis Laboratory's RBS Calculator which is generated as part of the RBS optimization.^34^

To reduce variability in the RBS function due to differences in the downstream genetic sequence, all peptide genes were expressed as a fusion of the Microcin V secretion tag (Vsp) and the mature bacteriocin. The sequence of Vsp was kept constant for all peptides and was used as the downstream sequence in the RBS optimization. We note that while further downstream regions will differ across peptides, it is the region proximal to the RBS that is thought to have the greatest impact on RBS function.^36^

### 2.2 Production of Enterocin A, Hiracin JM79, and Enterocin B

As mentioned above, we opted to use EcN as the delivery organism for antienterococcal bacteriocins. We thus transformed pMPES2 and pMPES2:A, B, H, and BHA into EcN to generate respectively a control strain, a strain producing Enterocin A, Enterocin B, and Hiracin JM79 individually, and a strain producing all three peptides. Enterocin A and Hiracin JM79 were selected because they were the most potent bacteriocins targeting VRE in the original pMPES system.^29^ Enterocin B was selected because of previous evidence that it may act synergistically or via a different mechanism of action with Enterocin A.^38^

**Figure 2:**
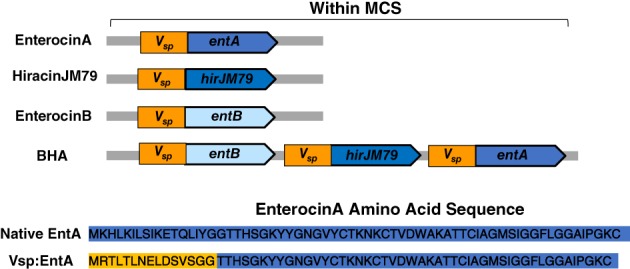
Genetic configuration for expression of bacteriocin genes. All bacteriocins were expressed as a fusion of the Microcin V secretion tag (Vsp) and the mature bacteriocin sequence. The resulting Enterocin A amino acid sequence is shown as an example

As mentioned above, all peptides were expressed as a fusion of the Vsp tag and the mature bacteriocin. The Vsp tag is thought to direct the secretion of the fused protein and is believed to be cleaved from the peptide upon secretion.^39^ Figure 2 depicts the Vsp fusions for the three peptides tested; Enterocin A, Hiracin JM79, Enterocin B, and an operon of the three peptides referred to as BHA.

After generating the bacteriocin constructs, we then tested the activity of the modified EcN strains against two vancomycin‐resistant clinical isolates, *Enterococcus faecium* 8E9 and *Enterococcus faecalis* V583R. *E. faecium* and *E. faecalis* were chosen because these two species are responsible for nearly all VRE infections.^40^ We note that the addition of pMPES2 or pMPES2 expressing the enterocins did not impact the growth rates or morphologies of any of the *E. coli* strains used (EcN, EcN RN, and *E. coli* MC1061 F′). In addition, plasmid stability was tested over 20 generations and no loss was observed.

**Figure 3:**
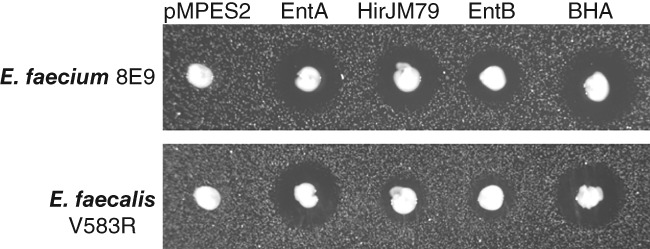
Agar diffusion assay showing Enterocin A, Hiracin JM79, and Enterocin B production from pMPES2 in EcN. Solid growth medium was seeded with *E. faecium* 8E9 and *E. faecalis* V583R then spotted with EcN producing no peptide (pMPES2), Enterocin A (EntA), Hiracin JM79 (HirJM79), Enterocin B (EntB), or all three peptides (BHA). White dot indicates EcN growth, light background indicates pathogen growth, and dark region is the zone of inhibition produced by EcN

Agar diffusion assays of the EcN strains against *E. faecium* 8E9 and *E. faecalis* V583R are shown in Figure 3. For these tests, *Enterococcus* was seeded in a solid growth medium plate then swabbed with the probiotic. The light background indicates pathogen growth, the white dot is the probiotic, and the dark region is the zone of inhibition produced by EcN.

Based on these initial screens, Enterocin A appeared to be the most potent individual peptide system. We note that halo diameter not only depends on the inhibitory concentration of the peptide itself, but also on the production and growth rates of the producer strain and the growth rate of the pathogen. Thus, we find halo diameters can typically be used to compare probiotic activities against a given target strain but should not be used to compare between different targets. We also note that at higher activity levels, halo diameters become more similar in size making quantitative comparisons of producer activity difficult.

To better quantify the overall probiotic activities, we performed EcN supernatant activity assays against *E. faecium* 8E9 and and *E. faecalis* V583R. For these studies, supernatant was collected from stationary phase EcN cultures and sterilized by filtration. Seven serial 2× dilutions of the sterile supernatant were then applied to *Enterococcus* cultures with a cell density of ~10^5^ colony forming units (CFUs)/ml and incubated overnight. Culture growths were monitored by optical density.

**Figure 4:**
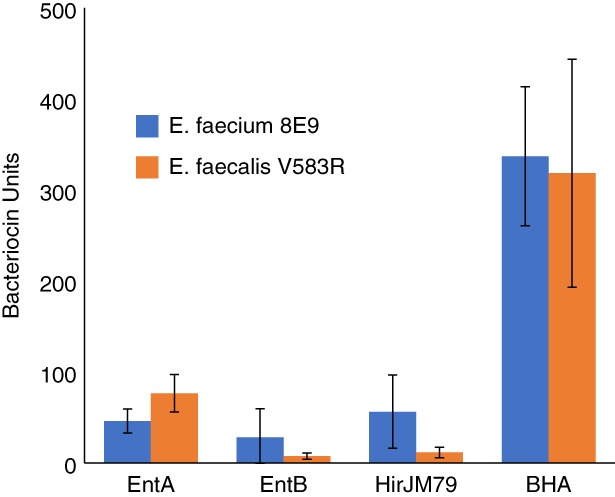
Relative supernatant activities from EcN producing Enterocin A (EntA), Enterocin B (EntB), Hiracin JM79 (HirJM79), or the combination of all three (BHA) against *E. faecium* 8E9 and *E. faecalis* V583R. Error bars indicate the SD across three biological replicates from independent experiments

Figure 4 shows the relative activities of EcN producing the three individual peptides and EcN producing the operon of three peptides. Herein, bacteriocin activity from the producer strains is quantified in terms of bacteriocin units (BUs).^41^ One BU is defined as the reciprocal of the highest dilution of the bacteriocin sample capable of inhibiting growth by 50%.

As expected from the agar diffusion assays, Enterocin A showed the highest level of activity among the individual peptides against *E. faecalis* V583R. Interestingly, EcN pMPES2:BHA showed a significantly higher activity than any of the three peptides independently. This difference in activity was not reflected in the agar diffusion assays; however, this is not unexpected because as mentioned above, halo sizes become more similar at higher activity levels.

The activity observed from EcN pMPES2:BHA was greater than the sum of the activities of the individual peptides against both pathogens. This may be due to synergistic activity of the peptides or due to an increase in production efficiency. It is not uncommon for operon gene expression to be more efficient than individual gene expression systems, largely due to improvements in translation efficiency.^42^ Several explanations have been posed for this increase in efficiency. It has been found that translation efficiency increases with the length of an RNA transcript, which is generally longer for polycistronic operons.^42^ In addition, it has been proposed that as the ribosome progresses along the upstream gene, it can denature otherwise inhibitory secondary structure in the mRNA in the downstream RBS regions.^43^, ^44^

### 2.3 Impact of operon organization

We next sought to test whether the order of the peptides in the operon would impact overall activity. This was done because it has been previously reported that operon efficiency can be drastically impacted by the order of the genes in an operon.^42^ Figure S2 (Supporting Information) shows the supernatant activities of pMPES2 with the six different peptide operons. As used in the BHA naming convention, H indicates Hiracin JM79, A is Enterocin A, and B is Enterocin B. The order of the three letters indicates the peptide order in the operon.

As anticipated, we observed different levels of activity from the six configurations. In particular, ABH showed significantly higher activity against *E. faecium* 8E9 than all other constructs except BHA (*P* < 0.05). Interestingly, this uniquely high activity was not observed against *E. faecalis* V583R. These results may indicate that the ABH configuration produced more peptides exhibiting stronger activity against *E. faecium* 8E9 compared to *E. faecalis* V583R. Importantly, HAB and ABH consistently exhibited numerically lower activities against both *E. faecium* 8E9 and *E. faecalis* V583R compared to all other constructs (*P* < 0.1). For example, ABH supernatant exhibited less than one third the activity against *E. faecium* 8E9 compared to BHA, HBA, and BAH supernatant and only one sixth the activity of ABH supernatant. These observations support the importance of operon configuration that is often overlooked.

Note that in Figure S2 (Supporting Information) a rifampicin and nalidixic acid‐resistant strain of EcN was used. We have found that both individual and multi‐peptide expression from this strain is significantly lower than from the wild‐type EcN (*P* < 0.05). This is based on a one‐tailed Student's *t* test assuming unequal variance (data not shown). This explains the inconsistent activity levels for BHA between Figure 4 and Figure S2 (Supporting Information). We selected this antibiotic‐marked strain for use in mouse studies to enable enumeration in the feces; however, in the future, we may explore alternative spontaneous mutants that do not exhibit hindered production.

### 2.4 Verification of peptide identity and activity

**Figure 5:**
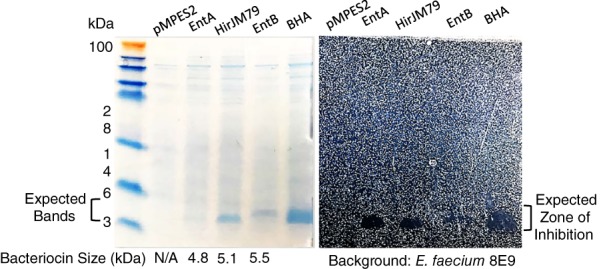
SDS‐PAGE of concentrated supernatant from EcN with pMPES2 (no AMPs) or pMPES2 expressing Enterocin A (EntA), Hiracin JM79 (HirJM79), Enterocin B (EntB), or all three peptides (BHA). Ammonium sulfate‐precipitated supernatant was run on an SDS‐PAGE gel, imaged, and then transferred to solid growth medium seeded with *E. faecium* 8E9. Left picture: Coomassie‐stained gel with bands at the expected size range for the peptides. Right picture: Zones of inhibition produced by the bands shown on the left

To verify that the anticipated peptides present in the supernatant were in fact produced and were the cause of activity, we performed sodium dodecyl sulfate–polyacrylamide gel electrophoresis (SDS‐PAGE) on the concentrated supernatants. Figure 5 shows the SDS‐PAGE gel of supernatants collected from cultures of EcN producing no bacteriocins (pMPES2) and EcN producing Enterocin A, Enterocin B, Hiracin JM79, or the operon of all three peptides (BHA) from pMPES2.

Clear bands were observed for Hiracin JM79, Enterocin B, and BHA at the expected size. It is interesting to note that only a faint band could be seen for Enterocin A despite it showing the greatest activity of the three individual peptides. The relative faintness in the Enterocin A band may partially result from the differences in amino acid sequences among the peptides. Coomassie dye binds proteins at positively charged amino acids including arginine and to a lesser extent lysine and histadine.^45^, ^46^ Both Enterocin B and Hiracin JM79 contain two arginine residues, four lysine residues, and one histidine residue. Enterocin A, however, contains five lysine residues but no histadine residues and most importantly, no arginine residues. Thus, one cannot readily compare the Enterocin A concentration to that of the other two peptides based on the SDS‐PAGE band intensity.

To verify that these bands were responsible for the observed antienterococcal activity, we overlaid the protein gel on solid growth medium containing *E. faecium* 8E9, similar to the agar diffusion assays presented above. From Figure 5, one can see zones of inhibition on the indicator strain over the supposed peptide bands.

As a final verification of their identities, the peptide bands were extracted from the gel using in‐gel trypsin digestion. The digested fragments were then analyzed by LC‐MS/MS on the Orbitrap Velos mass spectrometer by the University of Minnesota's Center for Mass Spectrometry & Proteomics. From these samples, we were able to detect the expected fragments corresponding to the trypsin‐digested peptides. We note that no quantitation was performed in these LC‐MS/MS studies. In future studies, we may explore absolute quantitation protocols via targeted LC‐MS/MS.^47^

### 2.5 Resistance prevention with the three‐peptide system

**Figure 6:**
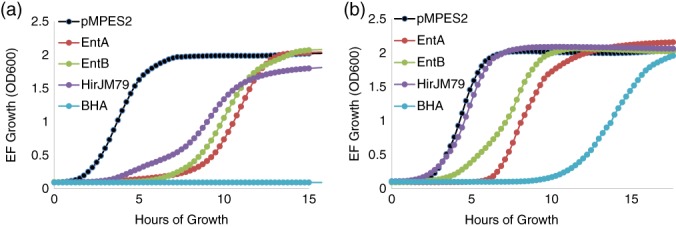
EcN supernatant activity against *E. faecium* 8E9 (A) and *E. faecalis* V583R (B). Cultures of *Enterococcus* were treated with serially diluted sterile supernatant from EcN producing no bacteriocins (pMPES2), Enterocin A (EntA), Enterocin B (EntB), Hiracin JM79 (HJirJM79), or all three peptides (BHA). For this figure, 12.5 vol/vol% supernatant is present in the culture. *E. faecium* growth was monitored by optical density at 600 nm. Data shown are from a single, representative experiment

In addition to drastically increasing overall activity, we have also observed that simultaneous production of the three enterocins reduces the development of resistance for *E. faecium* 8E9. Figure 6 shows the typical growth curves observed for *E. faecium* 8E9 and *E. faecalis* V583R treated with 12.5 vol/vol% supernatant from EcN cultures producing no bacteriocins (pMPES2) and EcN producing Enterocin A, Enterocin B, Hiracin JM79, or the operon of all three peptides (BHA) from pMPES2.

In all *E. faecium* cultures treated with individual peptides, there is strong inhibition of the pathogen for several hours followed by full regrowth. In the combination culture however, we observed no regrowth even after 48 hr of further incubation. In *E. faecalis* cultures, we did not see complete suppression of growth as we saw in *E. faecium* cultures. However, the three‐peptide system did drastically increase the time required for the pathogen to regrow compared to the individual peptides. We expect that this difference in behavior may be due to the lower activity levels of Enterocin B and Hiracin JM79 against *E. faecalis* compared to *E. faecium.* With greater peptide production, or with more potent peptides against *E. faecalis*, we anticipate that full inhibition could be achieved. Further studies are needed to verify this hypothesis.

Though our endpoint supernatant activities provide insight into the total peptide production over 17 hr for our probiotics, a more realistic study requires that the pathogen and probiotic compete within the same culture. We thus performed a coculture inhibition assay with EcN RN pMPES2:BHA and *E. faecium* 8E9 to determine if we could attain the same successful inhibition achieved in the supernatant assays. For this study, we inoculated *E. faecium* and EcN pMPES2 or pMPES2:BHA into the same culture and enumerated the number of surviving cells over time. Figure SI3 shows the *E. faecium* 8E9 counts (EF) and the EcN counts in cultures containing *E. faecium* alone, cultures containing *E. faecium* and EcN pMPES2, and cultures containing *E. faecium* and pMPES2:BHA.

From these results, it is clear that EcN RN pMPES2:BHA can compete with *E. faecium* 8E9 and that production of the three bacteriocins is able to eliminate *E. faecium* 8E9 without the emergence of resistance as seen in the supernatant inhibition assays. This lends promise to using this strain, despite its reduced activity compared EcN pMPES2:BHA.

Previously, we performed similar studies (both supernatant inhibition assays and coculture assays) involving Enterocin A, Hiracin JM79, and an additional peptide, Enterocin P.^48^, ^49^ In these studies, we consistently observed the same regrowth in *E. faecium* observed in the individual peptide cultures, regardless of the concentration of supernatant used. We found that the regrown population of *E. faecium* was stably resistant to the peptides. Enterocin A, Hiracin JM79, and Enterocin P are all class IIa bacteriocins and share the same extracellular protein target, a mannose phosphotransferase (ManPTS) (ManPTS).^49^ The disruption of this transporter apparently renders *E. faecium* resistant to all three peptides.

We hypothesize that the lack of resistance development against pMPES2:BHA may be due to the simultaneous application of peptides with orthogonal mechanisms of action and mechanisms of resistance. In this scenario, the pathogen would need to simultaneously develop two different resistant mutations. The probability of this occurring is orders of magnitude lower than for the development of a single resistant mutation.

**Figure 7:**
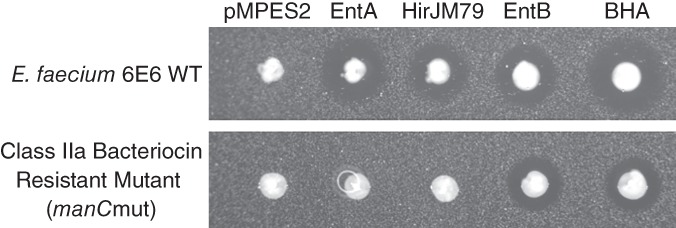
Orthogonality of Enterocin B activity to class IIa bacteriocins. The activities of EcN producing EntA, HirJM79, EntB, or all three was tested against *E. faecium* 6E6 wild type and *E. faecium* 6E6 ManPTS mutant. Enterocin A and Hiracin JM79 are inactive against the mutant while Enterocin B maintains activity. This suggests an orthogonal mechanism of action compared to the other two bacteriocins

It has been previously reported that Enterocin A‐resistant *E. faecalis* mutants remain susceptible to Enterocin B and that Enterocin B‐resistant *E. faecalis* mutants remain susceptible to Enterocin A.^38^ To determine if Enterocin B shows the same orthogonal mechanism of resistance against *E. faecium*, we performed agar diffusion assays of EcN producing the individual peptides and BHA using two strains of *E. faecium*; *E. faecium* 6E6 and a previously identified *E. faecium* 6E6 ManPTS mutant.^49^ Figure 7 shows the results of these activity assays.

One can see from these tests that while Enterocin A and Hiracin JM79 were inactive against the ManPTS mutant, Enterocin B and the three‐peptide construct maintained activity. This supports the hypothesis that the addition of Enterocin B may eliminate the class IIa bacteriocin resistant subpopulation in the culture leading to an overall reduced resistance development.

### 2.6 Studies in mice

We next sought to evaluate whether our probiotic could reduce VRE in the GI tract. To do this, we developed a VRE colonization model in mice, administered our engineered EcN in the water, and enumerated the VRE in the feces over time. In our colonization model, mice were administered ~5 × 10^8^ CFU/ml VRE (*E. faecium* 8E9 or *E. faecalis* V583R) in drinking water containing 250 μg/ml vancomycin for 8 days. Vancomycin was added because we had found it improved colonization stability.

After the colonization period, mice were provided with three different treatments in their water. The untreated group was given sterile water, the control group was given water containing ~5 × 10^8^ CFU/ml EcN RN pMPES2 (no bacteriocins), and the treated group was given water containing ~5 × 10^8^ CFU/ml EcN RN pMPES2:BHA. *E. faecium* 8E9 or *E. faecalis* V583R and EcN RN were enumerated in the feces for the duration of the experiment. *E. faecium* 8E9 experiments were terminated sooner than *E. faecalis* V583R experiments because *E. faecium* 8E9 counts in several mice had fallen below the limit of detection (~500‐1,000 CFU/g feces depending on fecal mass). These colonization trends are consistent with prior experiments we had performed when originally selecting the *E. faecium* and *E. faecalis* strains (data not shown).

**Figure 8:**
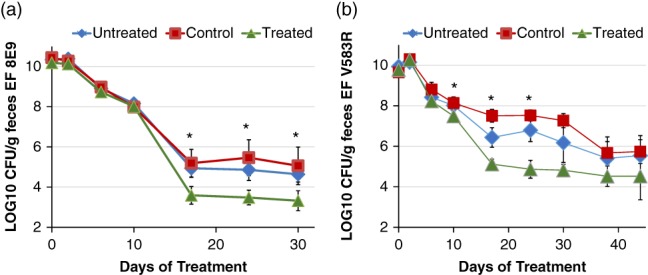
EcN RN reduction of *E. faecium* and *E. faecalis* in a murine model. Mice were colonized by *E. faecium* 8E9 (A) or *E. faecalis* (B) V583R via administration of 5 × 10^8^ CFU/ml in drinking water for 8 days. 250 μg/ml vancomycin was also added to the water to assist in colonization. On Day 9 (Day 0 of treatment), mice were administered sterile water (untreated), water containing 5 × 10^8^ CFU/ml EcN RN pMPES2 (control), or water containing 5 × 10^8^ CFU/ml EcN RN pMPES2:BHA (treated). *E. faecium* and *E. faecalis* (EF) were then enumerated in the feces for the duration of the experiment. Error bars represent the SE across mice within a treatment group (six mice per group)

Figure 8 shows the CFU/g feces of *E. faecium* 8E9 and *E. faecalis* V583R over time in the two separate experiments starting on the first day of probiotic administration. Water administration of EcN RN resulted in relatively stable concentrations of the probiotic in the feces for all mice. EcN RN was enumerated in the feces at each time point (Days 2, 6, 10, 17, 24, 30, as well as Days 38 and 44 for *E. faecalis* V583R). Average EcN RN pMPES2 counts throughout the experiment were 3.5x10^8^ CFU/g feces with a +/− SD range of 1.2 × 10^8^ to 1.0 × 10^9^ CFU/g feces. Average EcN RN pMPES2:BHA counts were slightly lower at 2.6 × 10^8^ CFU/g feces with a +/−SD range of 5.9 × 10^7^ to 1.1 × 10^9^ CFU/g feces. Ranges were calculated based on standard deviations of the log‐transformed CFU/g feces data across all mice in each group.

From Figure 8A,B, one can see that high colonization levels (~10^10^ CFU/g feces) of both *E. faecium* and *E. faecalis* were established by the end of the 8‐day colonization period. Once mice were no longer actively administered VRE in the water, these counts decline over time in all groups regardless of treatment. However, we observed that mice provided with EcN pMPES2:BHA in their water had significantly lower counts of both *E. faecium* and *E. faecalis* in their feces compared to both the untreated and control groups after approximately 2 weeks of administration.

We use two different statistical methods to evaluate this decrease; direct comparison of CFU/g feces on a day‐by‐day basis and a comparison of the area under the curve for CFU/g feces over time.^50^ The *p*‐values comparing CFU/g feces in the treatment group to those in the untreated and control groups for each day are provided in the Supplemental Information. These values are based on a one‐tailed Student's *t* test assuming unequal variance. Stars on Figure 8 indicates days on which the treated group was lower than the untreated group with *P* < 0.05.

The area under the CFU/g feces versus time curve was also calculated for each mouse and compared across groups. This area represents the total amount of VRE shed during the experiment. Based on this parameter, treated mice exhibited significantly less VRE in their feces over the duration of the experiment compared to either the untreated or control groups (*E. faecium*: untreated, *P* = 0.02; control, *P* = 0.03, *E. faecalis*: untreated, *P* = 0.031, control = 0.003).

We must note that the experiments above were done in male Balb/cJ mice. We then repeated the same tests for female Balb/cJ mice. With the exception of one outlier in the treated group, we saw similar reduction of *E. faecium* 8E9 in female mice as observed in male mice (Figure S4). However, we did not see this same reduction for the female *E. faecalis* V583R experiments (Figure S5). Interestingly, the control group behaved differently in these studies, suggesting an unknown variable may have been introduced. Additionally, the treated group had 100× higher *E. faecalis* levels at the end of the infection period, before the administration of EcN RN, than the other two groups. Based on these substantial discrepancies, we believe this *E. faecalis* trial should be repeated. We are now investigating what may have caused these colonization differences.

## 3 METHODS

### 3.1 Bacterial growth conditions

Bacteria and plasmids used in this study are reported in Table 1. Unless otherwise noted, *E. coli* was grown in lysogeny broth (LB) broth (Fisher Scientific, Fair Lawn, NJ) with agitation at 37 °C. Spectinomycin sulfate was added at 100 μg/ml for vector selection when necessary. *E. faecium* and *E. faecalis* were grown statically in brain heart infusion (BHI) (Oxoid Ltd., Basingstoke, United Kingdom) medium at 37 °C.

**Table 1:** Bacteria and plasmids used in this study

Strain	Description	Source
*E. coli* MC1061 F′	Plasmid‐free, recA+, nonamber suppressor cloning strain	Lucigen
*E. coli* Nissle 1917	Nonpathogenic human commensal used in the probiotic, Mutaflor	University of Minnesota
*E. coli* Nissle 1917 RN	Rifampicin and nalidixic acid resistant derivative of *E. coli* Nissle 1917	University of Minnesota
*E. faecium* 8E9	Ampicillin/vancomycin/linezolid resistant	University of Minnesota
*E. faecium* 6E6	Ampicillin/vancomycin/linezolid resistant	University of Minnesota
*E. faecium* 6E6 ManPTS mutant	*E. faecium* 6E6 derivative with a basepair deletion in ManC (mutant A4)	49
*E. faecalis* V583R	Rifampicin resistant derivative of *E. faecalis* V583	Medical College of Wisconsin

Plasmid	Description	Source
pHK22	pACYC184 derivative containing 9.4 kb MicV production fragment	University of Minnesota
pBFFusion	*E. coli* vector with synthetic promoter, ProTeOn+ upstream of green fluorescent protein (GFP)	University of Minnesota
pBFVIcolVsec	pBFFusionΔGFP replaced with Microcin V and containing a 3.6 kb MicV secretion system fragment from pHK22	University of Minnesota
pMPES2:CbnA	pBFVIcolVsecΔMicV replaced with Carnobacteriocin gblock with a modular molecular cloning site	This study
pMPES2	pMPES2:CbnA lacking CbnA (only molecular cloning site)	This study
pMPES2:A	pMPES2 with Enterocin A	This study
pMPES2:B	pMPES2 with Enterocin B	This study
pMPES2:H	pMPES2 with Hiracin JM79	This study
pMPES2:HAB	pMPES2 with operon of Hiracin JM79:Enterocin A: Enterocin B	This study
pMPES2:BHA	pMPES2 with operon of Enterocin B:Hiracin JM79: Enterocin A	This study
pMPES2:ABH	pMPES2 with operon of Enterocin A:Enterocin B: Hiracin JM79	This study
pMPES2:HBA	pMPES2 with operon of Hiracin JM79:Enterocin B: Enterocin A	This study
pMPES2:BAH	pMPES2 with operon of Enterocin B:Enterocin A: Hiracin JM79	This study
pMPES2:AHB	pMPES2 with operon of Enterocin A:Hiracin JM79: Enterocin B	This study

### 3.2 Construction of pMPES2

A 3.6 kb fragment containing the Microcin V secretion system was transferred from pHK22 into the *E. coli* expression vector, pBFFusion, thereby generating pBFVIcolVsec. To insert the molecular cloning site into pBFVIcolVsec, pBFVIcolVsec was first digested with SalI and PciI then purified with a Qiagen Qiaprep DNA purification kit (Qiagen, Hilden, Germany). The VspCbnA gblock (see Supporting Information) containing the molecular cloning site was then assembled into pBFVIcolVsec using New England Biolabs (NEB) Hifi Assembly Master Mix (New England Biolabs Inc., Ipswich, MA, USA) and transformed into *E. coli* MC1061 F′ (Lucigen, Middleton, WI). Successful pMPES2:CbnA transformants were then isolated and the vector was purified using the Qiaprep plasmid isolation kit (Qiagen). The carnobacteriocin A gene in pMPES2:CbnA was then removed by digesting the vector with SalI, purifying the digestion with Qiaprep kit, and transforming the digestion into *E. coli* MC1061 F′. A colony with a successfully digested vector resulting in pMPES2 was isolated and verified with Sanger sequencing.

### 3.3 Standard peptide insertion

All peptide DNA sequences, primers, and standardized parts mentioned below are provided in the Supporting Information. To insert the desired peptide fragment or fragments, pMPES2 was digested with SalI for single peptide insertions or SalI and NotI for triple peptide insertions (NEB). The digested vector was then purified with Qiagen Qiaprep DNA purification kit (Qiagen). Polymerase chain reaction with the appropriate assembly primers (first position: R_for/O_rev, second position: O_for/Y_rev, third position: Y_for/GB_rev) was performed to obtain peptide fragments with the necessary overhangs for assembly. We note that alternatively, we could have digested pMPES2 with SalI and AvrII and amplified the third position peptide with Y_for/G_rev as called for in the proposed protocol in the supplementary information. GB_rev was used for historical reasons based on previous protocols. The new workflow has been adapted for simplicity. Note all DNA templates are directly flanked by the RBS and 3′ PBS sequences. These standardized regions enable the overhang primers to bind. Original peptide fragments were ordered as gblocks from Integrated DNA Technologies.

### 3.4 In vitro activity assays

#### 3.4.1 Agar diffusion assays

*E. faecium* and *E. faecalis* were grown overnight in BHI medium. The indicated producer strain was streaked from a freezer stock onto LB agar with spectinomycin. The following morning, molten BHI agar (3.7 g BHI, 1 g agar per 100 ml) was prepared and allowed to cool to just above solidification temperature. The agar was inoculated with 0.5 μl overnight culture of the indicator strain per ml of agar, gently mixed by inversion, then poured into sterile petri dishes and allowed to solidify. The producer strains were then swabbed and stabbed into the plate. The plate was incubated overnight at 37 °C and imaged the following day.

#### 3.4.2 Supernatant activity assays

Enteroccocal strains were first streaked on BHI agar plates, and *E. coli* strains were streaked on LB with spectinomycin and incubated overnight. The following day, plates were used to start 2 ml overnight cultures of *Enterococcus* or 25 ml culture of *E. coli* in BHI medium and were incubated for ~17 hr. No antibiotics were added to any cultures for these studies. *E. coli* cultures were pelleted by centrifugation at 13,000*g* for 1 min. Supernatants were filtered using a 0.22‐μm filter (EMD Millipore, Billerica, MA) into sterile tubes and stored at 4 °C until use that same day.

For activity assays, 20 ml of BHI was inoculated with 2 μL of overnight *Enterococcus* culture to give ~10^5^ CFU/ml *Enterococcus.* A total of 125 μl of sterile supernatant was then added to the first row of wells of a sterile flat‐bottomed plate and 62.5 μl of sterile supernatant containing no AMPs was added to the remaining wells. Six 2× dilutions were performed down the rows and 187 μl of the *Enterococcus*‐inoculated BHI was added to all the wells giving final supernatant concentrations ranging from 25 to 0.39%. The plate was then covered and incubated for 20 hr at 37 °C with fast orbital shaking in a Synergy H1 plate reader (BioTek, Winooski, VT).

Supernatant activities are measured in BUs. One BU is defined as the reciprocal of the highest dilution of the bacteriocin that results in 50% growth reduction compared to the untreated culture.^41^ This was determined by first identifying the time at which the control culture transitioned to stationary phase (~1.9 in our assays). The OD600s of the growth curves of the treated cultures were then determined at this time point and the highest dilution with OD600 < 0.95 was determined. The dilution for a 50% reduction was then calculated by linear interpolation between these two dilutions and their resulting OD600s. P‐values were calculated using a one‐tailed Student's *t* test assuming unequal variance.

#### 3.4.3 Liquid coculture activity assay

*E. faecium* 8E9 and *E. coli* strains were inoculated separately from fresh plates into 2 ml BHI and incubated overnight at 37 °C for 18 hr. The following morning 50 μl of *E. faecium* 8E9 and 30 μl of *E. coli* were inoculated into 5 ml of fresh BHI and allowed to grow for 2 hr at 37 °C to enter exponential growth phase. The *E. coli* culture was centrifuged for 2 min at 2,000*g* and the pellet was resuspended in 5 ml fresh BHI to eliminate any peptides produced during the 2‐hr period. The *E. coli* and *E. faecium* 8E9 culture were then combined in a 1:1 ratio (150 μl of each) in wells of a 96‐well plate at *t* = 0 hr and incubated at 37 °C under static conditions.

Six 10× dilutions of a 10 μl sample of each culture were taken at each designated time point. Dilutions were then plated on LB + 150 μg/ml rifampicin + 30 μg/ml Nalidixic acid to enumerate *E. coli* and on m‐*Enterococcus* agar + 20 μg/ml Erythromycin to enumerate *E. faecium* 8E9. At 22 hr, 100 μl of the EcN BHA coculture was plated but no *E. faecium* was detected. We are thus claiming our limit of detection as 10 CFU/ml.

### 3.5 SDS‐PAGE and mass spectrometry verification

#### 3.5.1 Peptide concentration by AS precipitation

Fresh colonies of the producer strains were inoculated into 25 ml BHI medium and were incubated for ~17 hr at 37 °C. *E. coli* cultures were pelleted by centrifugation at 5,000*g* for 10 min and the supernatants were sterile filtered into to sterile tubes. Then, 11.25 g of ammonium sulfate salt was then added to each 25 ml sample to reach ~70% saturation concentration. Samples were mixed by rotation at 4 °C overnight. The following day, proteins were pelleted by centrifugation at 11,000*g* at 4 °C for 10 min. Supernatant was removed, and the pelleted precipitate was resuspended in 250 μl sterile deinonized water then stored at −20 °C.

#### 3.5.2 SDS‐PAGE

A total volume of 2 μl of the concentrated supernatants were combined with 2 μl sterile DI water, 1 μl NuPAGE Reducing Agent (10×), and 5 μl Novex Tris‐Glycine SDS Sample Buffer (2×) then heated at 85 ^°^C for 2 min in a thermalcycler. 10 μl samples were applied to Novex 16% tricine gel, 1.0 mm × 12 wells. Then, 10 μl of SeeBlue Plus2 Pre‐stained Protein Standard was applied to the outer wells. The gel was run at 125V until complete (~90 min). It was then rinsed in deionized water and stained with SimplyBlue SafeStain for 1 hr. The gel was then destained overnight and imaged the following day.

#### 3.5.3 Gel activity assay

The destained gel was imaged then placed using sterile tweezers on BHI agar seeded with *E. faecium* as described in the agar diffusion assay section. The plate was incubated overnight and was imaged the following day.

#### 3.5.4 In‐gel trypsin digest and mass spectrometry

Peptides were isolated from the SDS‐PAGE gel by in‐gel trypsin digest according to the protocol provided by the University of Minnesota's Center for Mass Spectrometry & Proteomics. Briefly, the peptide bands were excised from the gel and transferred to clean microcentrifuge tubes. Coomassie stain was removed by washing with 1:1100 mM ammonium bicarbonate:acetonitrile then were treated with acetonitrile. Gel pieces were reduced and alkylated in a series of washes and incubation steps first in 10 mM DTT in 50 mM ammonium bicarbonate, then in 55 mM iodoacetamide in 50 mM NH4HCO3, then in a 1:1 acetonitrile:100 mM ammonium bicarbonate solution, and finally in 100% acetonitrile.

Gel pieces were incubated in digestion buffer (50 mM NH4HCO3, 5 mM CaCl2, 5 ng/μl trypsin) at 4 °C. The digestion buffer then replaced with 70 μl 50 mM NH4HCO3, 5 mM CaCl2 and incubated overnight at 37 °C. The supernatant was removed then remaining peptide was extracted in a series of acetonitrile and formic acid resuspensions and collections. The pooled collections were then freeze‐dried and provided as samples to the Center for Mass Spectrometry & Proteomics.

Samples were then processed by LC‐MS/MS on the Orbitrap Velos mass spectrometer. The data were then interpreted by the software package PEAKS Studio 8.0.

### 3.6 Mouse studies

Eight week‐old Balb/cJ mice from Jackson Laboratories were used. All experiments shown here were performed at the University of Minnesota Research Animal Resources except the female Balb/cJ experiment using *E. faecalis* V583R. This experiment was performed at the Medical College of Wisconsin. Protocols were approved by the Institutional Animal Care and Use Committee and adhered to the institutional and national guide for the care and use of laboratory animals. Mice were housed three to a cage and were kept in autoclaved cages and provided autoclaved drinking water. Mice were allowed to acclimate for 2 days, at which point they were given drinking water containing ~5 × 10^8^ CFU EF/ml and 250 μg/ml vancomycin for 8 days (Day −7 to Day 0). Vancomycin was included in the drinking water because we have found it significantly improves the colonization stability of *E. faecium* 8E9.

On Day 0, mouse cages were randomly distributed into treatment groups with two cages to a group. The three treatment groups were as follows: untreated mice, control group, and treated group. Starting on Day 0, the untreated group received sterile water, the control group received water with 5 × 10^8^ CFU/ml EcN RN pMPES2 (produces no AMPs), and the treated group received water containing 5 × 10^8^ CFU/ml EcN RN pMPES2:BHA (produces Enterocin B, Hiracin JM79, and Enterocin A). The designated treatments were continued for the duration of the experiment.

Throughout the infection and treatment periods, water was exchanged every 3‐4 days to ensure consistent viability of both EcN and EF. Viability was also monitored at the start and end of each water exchange.

EcN RN and *Enterococcus* were enumerated in the feces of mice for the duration of the experiment. Mice were separated into plastic autoclaved boxes and allowed to sit for ~2‐5 min until approximately three fecal pellets were obtained. Fecal samples were transferred to sterile microcentrifuge tubes, massed, and stored at 4 °C for <5 hr.

1 ml of sterile PBS was added to each sample and samples were stored for an additional 30 min‐1 hr at 4 °C to loosen fecal pellets. Samples were vortexed for ~30 s to fully suspend feces. Six 10× serial dilutions were performed for each sample and dilutions were plated on the following selective growth plates; *E. faecium* 8E9: m‐*Enterococcus* agar + 20 μg/ml erythromycin; *E. faecalis* V583R: m‐*Enterococcus* agar + 150 μg/ml rifampicin; EcN: LB + 50 μg/ml spectinomycin + 150 μg/ml rifampicin + 30 μg/ml nalidixic acid. Plates were incubated overnight at 37 °C and colonies were counted the following day.

### 3.7 Statistical analysis

The aim was to compare the mean levels of VRE in mouse feces in the treated group versus the untreated group and the control group. Two methods were used; day‐by‐day comparison and comparison of the total VRE shed during the experiment (area under the CFU VRE/g feces curve). For both analyses, CFU VRE/g feces were first log‐transformed. To compare daily fecal counts across groups, a one‐tailed Student's *t* test was performed between either the treated and untreated or untreated and control groups assuming unequal variance. The treated group was considered statistically reduced for *p* < 0.05.

Total VRE in the feces over time was compared to provide a single, cumulative analysis of the observed reduction over time. To do this, the integral of the CFU VRE/g feces was approximated using the trapezoidal rule from Day 0 to the final day of the experiment. The mean areas for the six mice in each group were then compared using a one‐tailed Student's *t* test between either the treated and untreated or untreated and control groups assuming unequal variance. The treated group was considered statistically reduced for *P* < 0.05.

## 4 CONCLUSIONS

In this study, we have significantly improved upon a modular peptide expression system for EcN, making a viable probiotic delivery system to target pathogens in the intestinal tract. We have successfully shown that the engineered strain produces and secretes the peptides at sufficient levels to drastically inhibit pathogens of interest in laboratory conditions. We have also provided evidence suggesting that the simultaneous production of multiple peptides may help prevent the regrowth of resistance to bacteriocins. In future studies, we must examine how engineered probiotics may impact the host microbiome and better establish their robustness in female mice and other strains.

In this study, we have focused on the production of peptides targeting vancomycin‐resistant enterococci. However, because the secretion system used for pMPES2 is widely applicable for different peptides, we can readily modify our probiotic for use against a variety of Gram‐positive and Gram‐negative pathogens. Though further improvement and analysis will need to be done to achieve a clinical product, the *in vitro* and *in vivo* results presented here provide strong proof of concept evidence for pMPES2 as a probiotic‐based bacteriocin delivery vector.

## CONFLICT OF INTEREST

The authors declare that they have no conflicts of interest with the contents of this article.

## Supporting information

Supporting Information
