# Supplementary material for: Engineered E. coli Nissle 1917 for the reduction of vancomycin‐resistant Enterococcus in the intestinal tract
Source: Bioeng Transl Med. 2018 Sep 8;3(3):197–208. doi: 10.1002/btm2.10107 (PMC6195901; doi:10.1002/btm2.10107)
Supplement: Supplementary file 1 — Supporting Information [file BTM2-3-197-s001.docx]

**Supporting Information**


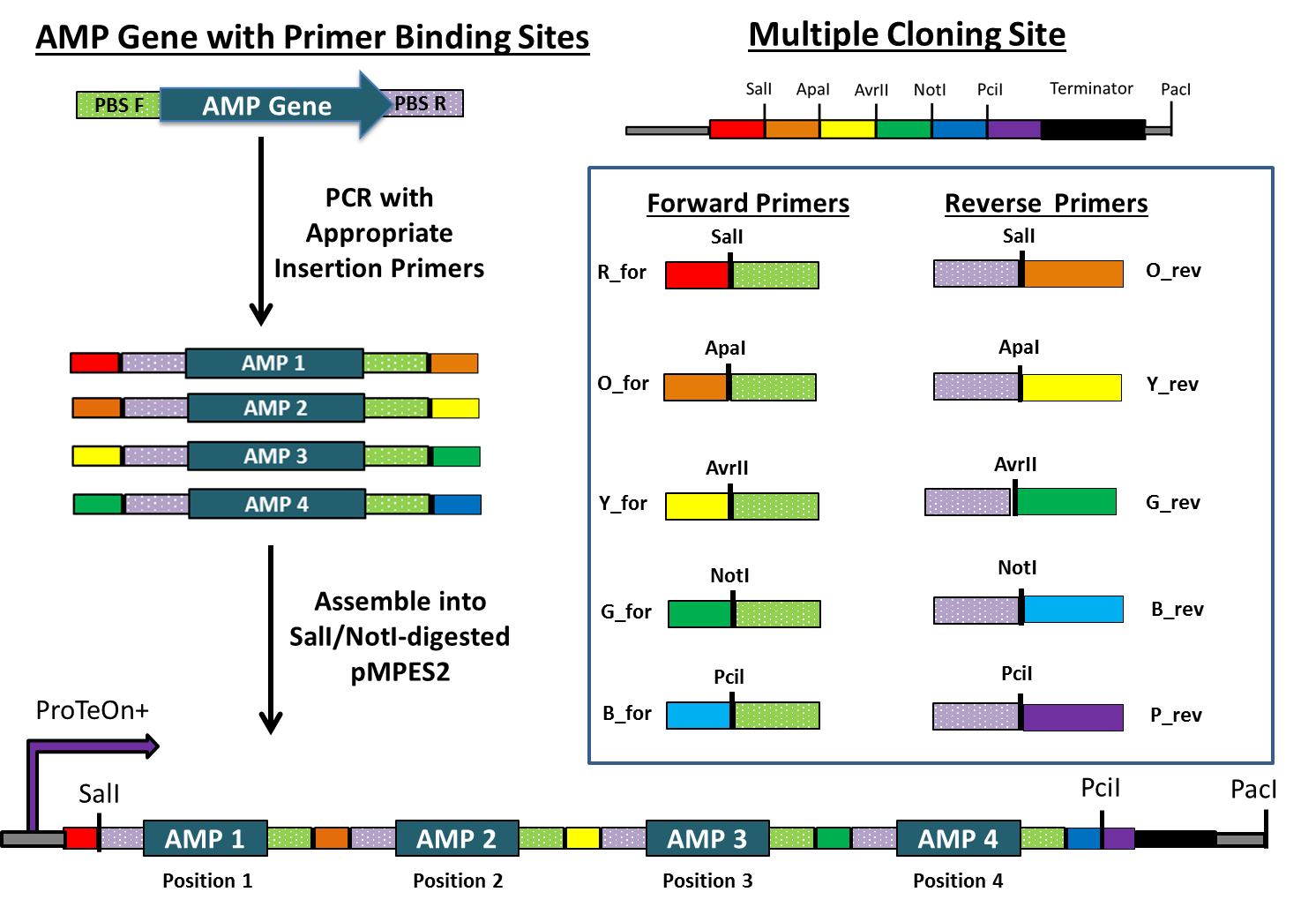


**Figure SI1. Modular AMP cassette assembly using the pMPES multiple cloning site.** Molecular cloning site contains five restriction enzymes cute sites followed by a terminator sequence. Between each cut site is an “overlap region” which is used with the forward and reverse primers shown to enable assembly of the final construct. AMPs and their immunity genes will be synthesized to include the primer binding sites PBS F and PBS R. The appropriate forward and reverse primers are then selected and fused to the AMP via PCR to attach the necessary overlap region. Reverse primers also introduce an additional restriction site following each AMP (identical to that found in the forward primer) to later allow seamless removal of individual AMPs from the assembly. AMPs with their attached overlap regions are then assembled with the linearized pMPES2 backbone to give the final construct.

**Examples for Modular Assembly Workflow:**

**Single Peptide Insertion**

Into Position 1: Digest pMPES2 with SalI, amplify AMP 1 with R_for/O_rev

**Double Peptide Insertion**

Digest pMPES2 with SalI and ApaI, amplify AMP 1 with R_for/O_rev; amplify AMP 2 with O_for/Y_rev

**Triple Peptide Insertion**

Digest pMPES2 with SalI and AvrII, amplify AMP1 with R_for/O_rev; amplify AMP 2 with O_for/Y_rev; amplify AMP3 with Y_for/G_rev

**
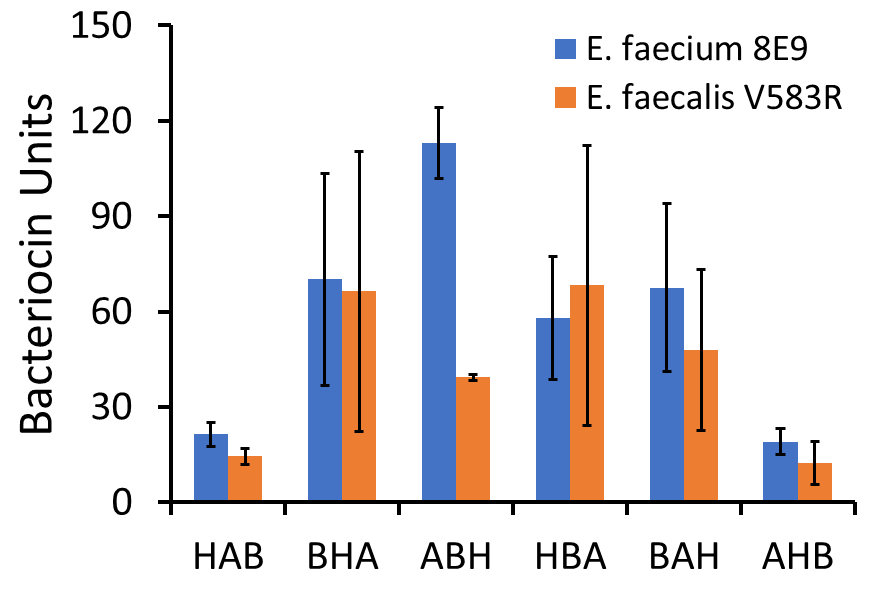
**

**Figure SI2. Relative supernatant activities from EcN RN producing Enterocin A (A), Enterocin B (B), and Hiracin JM79 (H) from different operon configurations against E. faecium 8E9 and E. faecalis V583.** Error bars indicate the standard deviation across three biological replicates from independent experiments. Order of letters indicates order of peptides in the operon.


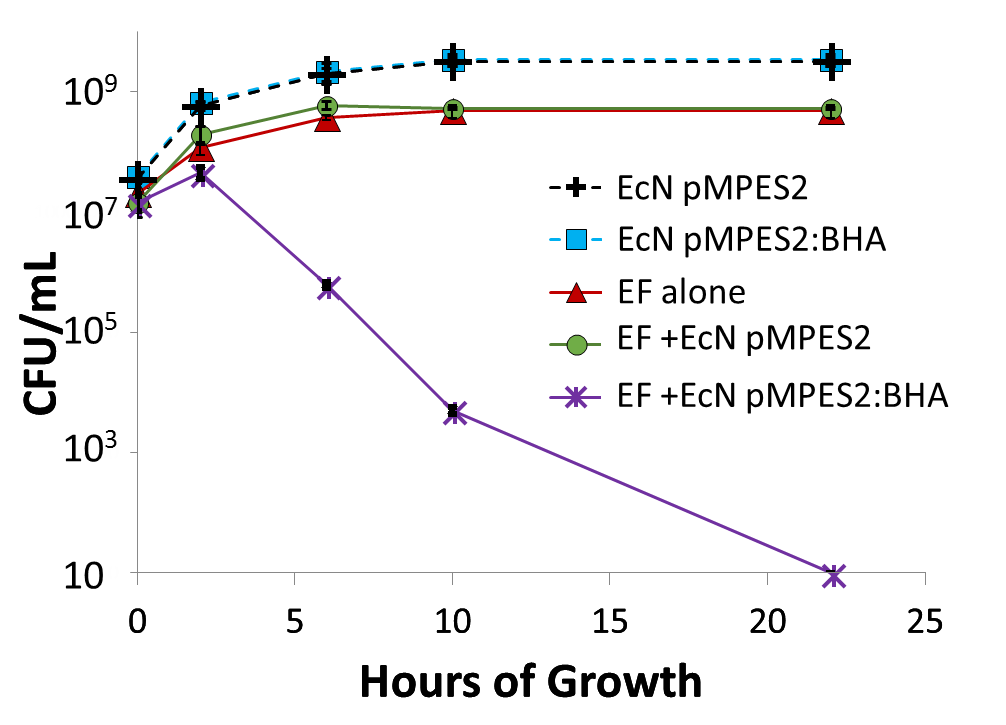


**Figure SI3. Co-culture of engineered EcN RN and *E. faecium* 8E9.** A culture of 10^7^ CFU/mL *E. faecium* 8E9 was grown alone or inoculated with 10^7^ CFU/mL EcN RN containing either pMPES2 (no AMPs) or pMPES2:BHA (expressing Enterocin B, Hiracin JM79, and Enterocin A. EcN RN and *E. faecium* (EF) were enumerated in all three cultures over time. EcN RN and *E. faecium* 8E9 counts obtained from co-cultures are both presented in the figure. Note no resistant subpopulation was observed among *E. faecium* 8E9. Error bars represent standard deviation of biological triplicates from a single experiment.

**
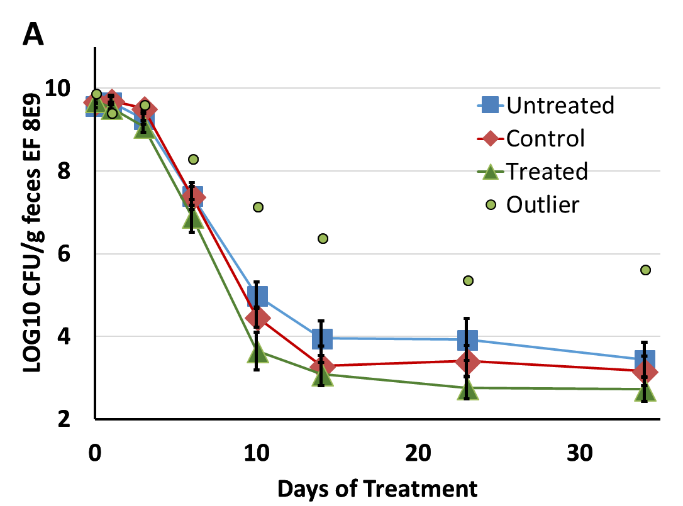

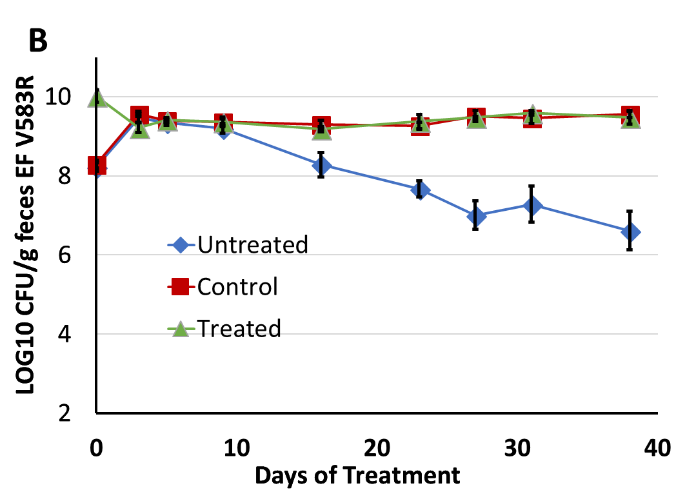
**

**Figure SI4. Testing EcN RN Efficacy Against *E. faecium* and *E. faecalis* in a female Balb/cJ murine model.** Mice were colonized by *E. faecium* 8E9 (a and b) or *E. faecalis* V583R via administration of 5x10^8^ CFU/mL in drinking water for 8 days. 250 ug/mL vancomycin was also added to the water to assist in colonization. On day 9 (day 0 of treatment), mice were administered sterile water (Untreated), water containing 5x10^8^ CFU/mL EcN RN pMPES2 (Control), or water containing 5x10^8^ CFU/mL EcN RN pMPES2:BHA (Treated). *E. faecium* and *E. faecalis* (EF) were then enumerated in the feces for the duration of the experiment. Error bars represent the standard error across mice within a treatment group (six mice per group). Outliers were determined using the Grubbs’ Test for Outliers with p < 0.05 performed on area under the curve data.

**P-Values in Figure S14**

Figure S14. *E. faecium* 8E9 CFU/g feces

Individual Days

| **TTEST results** | **day 10** | **day 14** | **day 23** | **day 34** |
| --- | --- | --- | --- | --- |
| Treated vs. Untreated | 0.022 | 0.061 | 0.039 | 0.101 |
| Treated vs. Control | 0.081 | 0.368 | 0.100 | 0.182 |

Area Under the Curve

| **TTEST results** |  |
| --- | --- |
| Treated vs. Untreated | 0.011 |
| Treated vs. Control | 0.098 |

**P-Values in Figure 8**

Figure 8a. *E. faecium* 8E9 CFU/g feces

Individual Days

| **TTEST results** | **day 10** | **day 17** | **day 24** | **day 30** |
| --- | --- | --- | --- | --- |
| Treated vs. Untreated | 0.310 | 0.030 | 0.032 | 0.035 |
| Treated vs. Control | 0.451 | 0.041 | 0.041 | 0.071 |

Area Under the Curve

| **TTEST results** |  |
| --- | --- |
| Treated vs. Untreated | 0.020 |
| Treated vs. Control | 0.029 |

Figure 8b. *E. faecalis* V583R CFU/g feces

Individual Days

| **TTEST results** | **day 10** | **day 17** | **day 24** | **day 30** | **day 38** | **day 44** |
| --- | --- | --- | --- | --- | --- | --- |
| Treated vs. Untreated | 0.048 | 0.022 | 0.012 | 0.115 | 0.237 | 0.264 |
| Treated vs. Control | 0.051 | 0.000 | 0.001 | 0.000 | 0.082 | 0.200 |

Area Under the Curve

| **TTEST results** |  |
| --- | --- |
| Treated vs. Untreated | 0.031 |
| Treated vs. Control | 0.003 |

**DNA Sequences**

| **Modular DNA Sequences in Figure 2** | |
| --- | --- |
| Red Overlap | ATTTCAAAAACTGAGAATTCGTCGAC |
| Orange Overlap | CCACAGTAGTTACAGAGGGCCC |
| Yellow Overlap | CTGCAGCTATAGCGACCCTAGG |
| Green Overlap | CCTAGAACATATAGCGGCCGC |
| Blue Overlap | CCATCACACTGTGAGCACATGT |
| Purple Overlap | CCGTAGCTAGCGAAAAAAAAACCCGC |
| Ribosomal Binding Site (RBS) | TACATAACAACAGCAACAACTAAGGAGGTTTTCA |
| Primer Binding Site (PBS) | GGTCGAAGCTCAGAGGATCGTACAG |

**Primers for Peptide Insertion (RBS and PBS are Underlined)**

| **Primer Name** | **DNA Sequence** |
| --- | --- |
| R_for | ATTTCAAAAACTGAGAATTCGTCGACTACATAACAACAGCAACAACTAAGGAGGTTTTCA |
| O_rev | GGGCCCTCTGTAACTACTGTGGGTCGACCTGTACGATCCTCTGAGCTTCGACC |
| O_for | CCACAGTAGTTACAGAGGGCCCTACATAACAACAGCAACAACTAAGGAGGTTTTCA |
| Y_rev | CCTAGGGTCGCTATAGCTGCAGGGGCCCCTGTACGATCCTCTGAGCTTCGACC |
| Y_for | CTGCAGCTATAGCGACCCTAGGTACATAACAACAGCAACAACTAAGGAGGTTTTCA |
| GB_rev | ACATGTGCTCACAGTGTGATGGGCGGCCGCTATATGTTCTAGGCCTAGGCTGTACGATCCTCTGAGCTTCGACC |

**VspCbnA Gblock:**

GTCAGAGCATTAGCAAAGCAATTCTGACCGGTGCCATTGATTGTCCGTATTTCAAAAACTGAGAATTCgtcgacTACATAACAACAGCAACAACTAAGGAGGTTTTCA***atgcgcactctgactctgaatgaattagattctgtttctggtggt****gaccagatgtctgatggggtcaactatggcaaggggtctagtctgtctaaaggcggagcgaaatgtggtttgggtattgtcggaggtctggcgacaattccctctggacctttaggatggttagctggtgcggctggcgttatcaactcttgtatgaagtaa*GGTCGAAGCTCAGAGGATCGTACAGGTCGACCCACAGTAGTTACAGAGGGCCCCTGCAGCTATAGCGACCCTAGGCCTAGAACATATAGCGGCCGCCCATCACACTGTGAGCACATGTCCGTAGCTAGCGAAAAAAAAACCCGC

*RBS and PBS are underlined, VspCbnA is in lowercase italics with Vsp in bold

**Bacteriocin DNA Sequences**

**Enterocin A: atgcgcactctgactctgaatgaattagattctgtttctggtggt**accactcatagcggtaagtattacggaaatggagtttactgtaccaaaaataaatgcaccgttgattgggctaaagcgacaacttgtatcgctggtatgtctatcggcgggttcttagggggtgccattccaggcaaatgctaa

**Enterocin B:**

**atgcgcactctgactctgaatgaattagattctgtttctggtggt**gaaaacgaccacagaatgcccaacgagttgaatcgccctaacaatcttagcaaagggggagccaaatgcggcgcggcgattgcaggtggacttttcgggataccgaaaggaccgctggcttgggccgctggattagcgaatgtttactcaaaatgtaactaa

**Hiracin JM79:**

**atgcgcactctgactctgaatgaattagattctgtttctggtggt**gcgacatactatggaaatggattgtattgcaataaggagaagtgttgggtcgattggaatcaagctaaaggagagatcggaaagataatagtgaacggttgggtgaatcatggtccttgggctcccagacgctaa
